# Vocal Fold Cancer Transoral Laser Microsurgery Following European Laryngological Society Laser Cordectomy Classification

**DOI:** 10.3389/fonc.2018.00231

**Published:** 2018-06-22

**Authors:** Abie H. Mendelsohn, Marc Joseph Remacle

**Affiliations:** ^1^Department of Head and Neck Surgery, UCLA David Geffen School of Medicine, Los Angeles, CA, United States; ^2^Department of Otorhinolaryngology Head and Neck Surgery, Centre Hospitalier de Luxembourg – Clinique d’Eich, Luxembourg, Luxembourg

**Keywords:** laser cordectomy, glottic cancer, voice, endoscopy, KTP

## Abstract

**Background:**

The surgical treatment of glottic, or vocal cord, cancer has seen considerable progression over the past several decades. Specifically, there has been a stark transition from open partial laryngectomy surgery to endoscopic laser microsurgical techniques which have been inspired in large part by two landmark studies: Professor Wolfgang Steiner’s original case series describing transoral laser microsurgery for glottic cancer (1993) and the European Laryngological Society’s (ELS) classification scheme (2000). We performed a comprehensive review of published literature to characterize the pattern of this novel modality as compared with two landmark studies over the past four decades.

**Methods:**

An English literature search was conducted on PubMed for available original investigations on surgical treatment of glottic laryngeal cancer published over the past 40 years. Our Boolean criteria included the following terms: cancer, glottic, laryngeal, surgery, endoscopic, and laser. The publication rates were calculated as annual compound growth as well as corrected growth rates as defined by the Fisher equation for inflation effects.

**Results:**

Our review identified 13,372 studies covering larynx cancer and 3,557 studies covering glottic cancer original studies. Among these, we analyzed the compound annual growth rates and correct growth rates for three distinct publication periods or epochs, prior to 1993, 1993–1999, and 2000–2017. For all but two of the search term groups covering both glottic cancer as well as larynx cancer, there was a substantial growth improvement in the time period following the ELS classification scheme as compared with the growth rate of the time period following Steiner’s case series.

**Conclusion:**

The progress toward minimally invasive treatment of glottic cancer has progressed steadily over the past several decades. Analysis of publication show increased growth during the time period following the ELS classification scheme over the time period following Steiner’s landmark study. A mistake would be concluding any diminished importance of Professor Steiner’s work, instead, our analysis demonstrates the wide-spread adoption of the endoscopic laser cordectomy procedure following the ELS classification system. Complex surgical techniques such as transoral laser microsurgery are optimally disseminated within well-defined classification schemes, though further validation is warranted.

## Introduction

Vocal fold cancer, particularly early staged (T1–T2) glottic cancer, is recognized to have reliably excellent cure rates irrespective of treatment modality (1–3). Specifically, local control and laryngectomy-free survival exceeds 90% following primary radiotherapy or definitive surgical resection. With the oncologic success rates nearly optimized, treatment advances have been motivated to minimize treatment-related morbidity thereby optimizing functional outcomes. External beam radiation advances have been shown with the adoption of intensity modulated radiation therapy (IMRT) which reduced the size and treatment volume. With less of the pharyngolarynx impacted by the effects of radiation, IMRT has been thought to improve voice and swallowing morbidity over conventional radiotherapy designs (4). Similarly, primary surgical strategies have looked to optimize functional outcomes mainly by transitioning oncologic resection from open cervical surgery to transoral endoscopic approaches. Minimizing the area of larynx which is affected by surgery has been shown to optimize airway management as well as post-surgical voicing (5).

The transition from open surgical resection to endoscopic resection has been seen over the past 40 years. As early as the 1970s, Strong and Jako adapted the CO_2_ laser for endoscopic laryngeal surgery (6). Despite this critical advance in technologic capacity, generally it is Professor Wolfgang Steiner who was attributed with the popularization of the CO_2_ laser endoscopic resection of glottic cancers. In 1993, Steiner published the seminal work describing his considerable experience and results for endoscopic CO_2_ laser resection of glottic cancers (7). Steiner’s approach was revolutionary, bucking the dogma of oncology surgeons of the time who demanded en-bloc tumor resections. Instead, Steiner described a stepwise process toward piecemeal removal of the glottic tumors. As Steiner argued, a surgeon could use the CO_2_ laser to bisect the tumor, thereby establishing the deep oncologic margin, and subsequently resect the tumor areas anterior and posterior to this bisecting cut. In this seminal manuscript, Steiner reported excellent and reliable oncologic outcomes with preservation of laryngeal function. However, anecdotally, many surgeons world-wide argued against Steiner’s approach on the basis of inability to reproduce the reported success rates with the technique.

Following Steiner’s popularization of the approach, the endoscopic CO_2_ laser surgery for glottic cancers continued to progress slowly until a landmark manuscript by the collaborative work of the European Laryngological Society (ELS), comprised of the leading European authorities in laryngeal cancer surgery. The statement manuscript called for a unified classification scheme for endoscopic CO_2_ laser resection of glottic cancer, otherwise termed CO_2_ laser cordectomy. One of the primary goals of the ELS consensus statement was to create a uniform language for outcomes comparison across intuitions and manuscripts. Another critical goal of the ELS system was to define a reliably reproducible systematic approach toward endoscopic resection which would be defined by the invasiveness and areas involved with tumor growth (8, 9). Therefore, with the establishment of the ELS cordectomy classifications much of the judgment calls and reliance on clinical experience needed with Steiner’s approach was improved. The ELS laser cordectomy system proposed a sequential protocol that surgeons could utilize based on their assessment of the extent of cancer. Specifically, bulky unilateral tumors might be treated with an *intramuscular cordectomy* (ELS type III), whereas deeply invasive tumors might be better addressed *via* a *complete cordectomy* (ELS type IV).

As such, it has been suggested that the ELS classification system succeeded in not only offering a common language to compare surgical results but more importantly offering specific surgical specifications for the spectrum of glottic invasive carcinoma. But this suggestion has yet to be evaluated or confirmed. Some investigators have studied the evolving treatment patterns of laryngeal cancer through national database analyses, yet the use of central data-banks hold significant limitations and assumptions which limit their ability to answer our question. Therefore, we sought to analyze the impact of the ELS Cordectomy classification on the evolution of endoscopic laser resection of glottic cancer by analyzing peer-reviewed medical publication rates.

## Materials and Methods

The historical literature citations of the two landmark studies of interest were analyzed. To perform this analysis, the manuscripts were searched within the *Web of Science Core Collection Database* which provides comprehensive citation count and analysis. (http://apps.webofknowledge.com/WOS_GeneralSearch_input.do?product=WOS&search_mode=GeneralSearch&SID=7AiwMkTvq28ge7aOYLA&preferencesSaved=, accessed May 27, 2018) However, following this analysis it was determined that no conclusion could be made based on the comparative impact on the field of glottic cancer.

We, therefore, sought to identify the rate of publications regarding the topic of endoscopic resection of glottic cancer. A literature search was conducted on PubMed for available English Language publications on the subject of interest. All articles were recorded initially, but were then limited based on the publication year of 1975. This limit was chosen to offer comparative publication total for comparisons across all search term groups, as several search groups totaled zero prior to 1975. The search was performed in January 2018, therefore, publication year of 2017 was included while publication year of 2018, having only just started, was excluded. Therefore, all of our search term groups were limited between 1975 and 2017.

The search term groups were performed with two base search titles. The first round of search groups was done using the titles of both “GLOTTIC” and “CANCER.” The second round of search groups was done using the titles of both “LARYNX” and “CANCER.” Both search titles were then redefined by more specific Boolean criteria which added to the base titles the following terms: *surgery*, then including *endoscopic* and/or *Laser* (all performed individually as opposed to a single Boolean search term). Once the search was completed, we computed the annual manuscript volumes. Annual manuscript volumes were then converted into cumulative growth totals through standard Excel (Volume 15.32, Microsoft, Seattle, WA, USA) formulation.

Our goal was to compare the effects that the two major papers in the field of endoscopic laser treatment for glottic cancer on publication rates. Therefore, our searches were divided into three distinct time periods, or eras. The first period was defined as “before Dr. Steiner’s landmark publication” (7), or 1980–1992 (with cumulative total for beginning total = publications from 1975–1980). Notably, though the literature search included manuscripts from 1975 and on, the first group was defined as from 1980 and on in order that the baseline growth rate would be representative of the manuscripts published in the 5 years prior to the group start date. Starting the analysis with a previous annual total of zero creates an extremely skewed measure of publication growth. The second period was defined as “after Dr. Steiner’s publication but before the ELS cordectomy classification,” or 1993–1999. The third period was defined as “after ELS classification proposal,” (8) or 2000–2017.

We then took the previously calculated cumulative totals and then converted these into a compound annual growth rate (CAGR). The formula used for CAGR is well described in the economics field:

$$\text{CAGR}={\left(\text{Ending Value}/\text{Beginning Value}\right)}^{\left(1/\text{number of years}\right)}-1.$$

By converting the cumulative totals into the CAGR, we would adjust for the varying overall number of years in each of the three defined time periods.

However, a comparison between the time period growth rates at this point offers inaccurate comparison as the overall change in medical journal scholarship and publications which is expected when looking over a long time period such as this. Therefore, a correction was required which could adjust for this overall publication growth rate. If we are to accept that there is regular increase in medical publications over the years, we may describe this overall growth rate as publication “inflation.” To address the effect of publication inflation, we return to the field of economics. The Fisher equation in financial mathematics and economics estimates the relationship between nominal and real interest rates under inflation. Which, after derivations holds true to the initial equation of (*r* ≈ *i−*π), where *r* = real growth rate, *i* = nominal growth rate, π = inflation rate.

Therefore, as our primary study goal is the analysis of the growth of publications describing endoscopic laser surgery for glottic (or laryngeal) cancer, we can define the overall growth rate of manuscripts within the field as manuscripts related to the title search terms of “glottic (or laryngeal) cancer,” without mention of surgery or laser. Therefore, we can define the growth rate of manuscripts published under the search terms glottic (or laryngeal) and cancer as a so-called inflation rate (π). We can then use the growth rate of the specific terms of endoscopic and/or laser combined with the baseline terms of glottic (or laryngeal) and cancer as the rate we measure as the nominal growth rate (*i*). Thus, to determine the real growth rate (*r*) of manuscript publications within the term of interest we will subtract the inflation rate from the nominal growth rate (*r* ≈ *i−*π).

## Results

**Figure 1:**
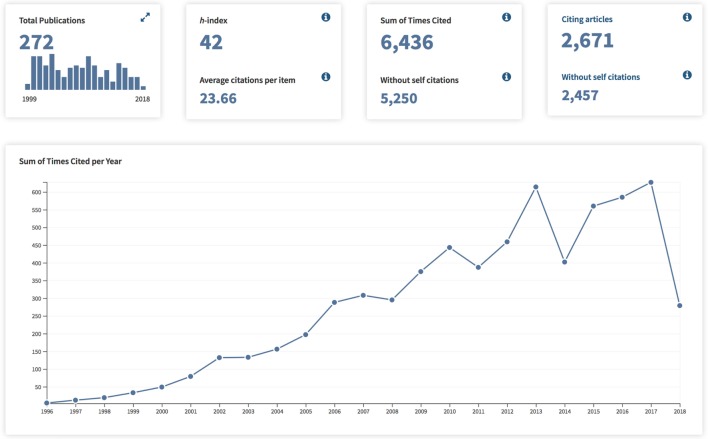
Publication Citation Analysis for “Results of curative laser microsurgery of laryngeal carcinomas” (Web of Science).

**Figure 2:**
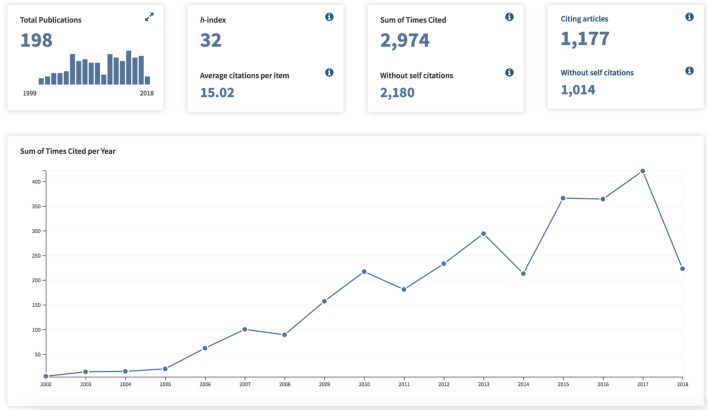
Publication Citation Analysis for “Endoscopic cordectomy. A proposal for a classification by the Working Committee, European Laryngological Society” (Web of Science).

We sought to evaluate the effects on the medical scholarship of the two landmark studies which have help shape the field of transoral laser microsurgery for glottic cancer. We first compared the growth rates to the *Web of Science* citation trends as displayed in Figures 1 and 2. Both of the publications of interest in this analysis demonstrate continuously increasing citations throughout the years following initial publication. The study by Steiner demonstrates over double the total number of citing articles (2,671 as compared with 1,177 for the ELS classification scheme), as well as a substantially increased Sum of Times Cited (6,436 as compared with 2,974 for the ELS classification scheme). However, what we see is that based on the aggregate time line, the vast majority of Times Cited occur within the most recent 7 years which is the interval time span between the two publications. Therefore, additional analysis was required to evaluate the publication effects of the two articles of interest.

We, therefore, proceeded to evaluate the publication growth rate within three distinct time periods as related to the publication years of the landmark manuscripts defining the CO_2_ laser endoscopic resection of early glottic cancer. Cumulative totals for English Language publications for “GLOTTIC and CANCER” between 1975 and 2017 was 3,557. Cumulative totals for English Language publications for “LARYNX and CANCER” between 1975 and 2000 was 13,372. Tables 1 and 2 delineate both the CAGR of the publications as well the corrected annual growth rates using the title search term of glottic as well as larynx. Generally, the time period before 1993 saw both large annual publication growth rates for glottic terms as well as larynx terms. Though the uncorrected annual growth rates for the periods of 1993–2000 and 2000–2017 showed consistent publication increases, when evaluating the growth rate specifically for the surgical management of glottic/larynx cancer the corrected values diminish significantly.

**Table 1:** Publication rates for glottic cancer.

	Compound annual publication growth rate			Real (or corrected) annual publication growth rate		
	1980–1992	1993–1999	2000–2017	1980–1992c	1993–1999c	2000–2017c
Glottic and cancer	9.37%	6.60%	4.44%	–	–	–
Glottic and cancer and surgery	9.51%	6.54%	4.96%	0.14%	−0.06%	0.52%
Glottic and cancer and surgery and laser	20.57%	8.30%	6.85%	11.20%	1.70%	2.40%
Glottic and cancer and surgery and endoscopic	11.75%	9.48%	7.13%	2.38%	2.87%	2.69%
Glottic and cancer and surgery and endoscopic and laser	27.06%	7.89%	6.96%	17.69%	1.29%	2.51%

*The compound annual growth rate is calculated for three distinct time periods: prior to Steiner’s landmark publication (1980–1992), after Steiner’s landmark publication until European Laryngological Society (ELS) Cordectomy Classification (1993–1999), and after the ELS Cordectomy Classification (2000–2017). Also shown are the corrected growth rates by adjusting the surgical publication rates based on overall growth rates within the topic of glottic cancer*.

**Table 2:** Publication rates for larynx cancer.

	Compound annual publication growth rate			Real (or corrected) annual publication growth rate		
	1980–1992	1993–1999	2000–2017	1980–1992c	1993–1999c	2000–2017c
Larynx and cancer	11.27%	6.52%	4.44%	–	–	–
Larynx and cancer and surgery	10.49%	6.86%	5.46%	−0.78%	0.34%	0.92%
Larynx and cancer and surgery and laser	17.03%	7.43%	5.97%	5.76%	0.91%	1.42%
Larynx and cancer and surgery and endoscopic	11.96%	7.95%	7.34%	0.70%	1.43%	2.80%
Larynx and cancer and surgery and endoscopic and laser	22.23%	7.22%	6.60%	10.97%	0.69%	2.05%

*The compound annual growth rate is calculated for three distinct time periods: prior to Steiner’s landmark publication (1980–1992), after Steiner’s landmark publication until European Laryngological Society (ELS) Cordectomy Classification (1993–1999), and after the ELS Cordectomy Classification (2000–2017). Also shown are the corrected growth rates by adjusting the surgical publication rates based on overall growth rates within the topic of larynx cancer*.

**Figure 3:**
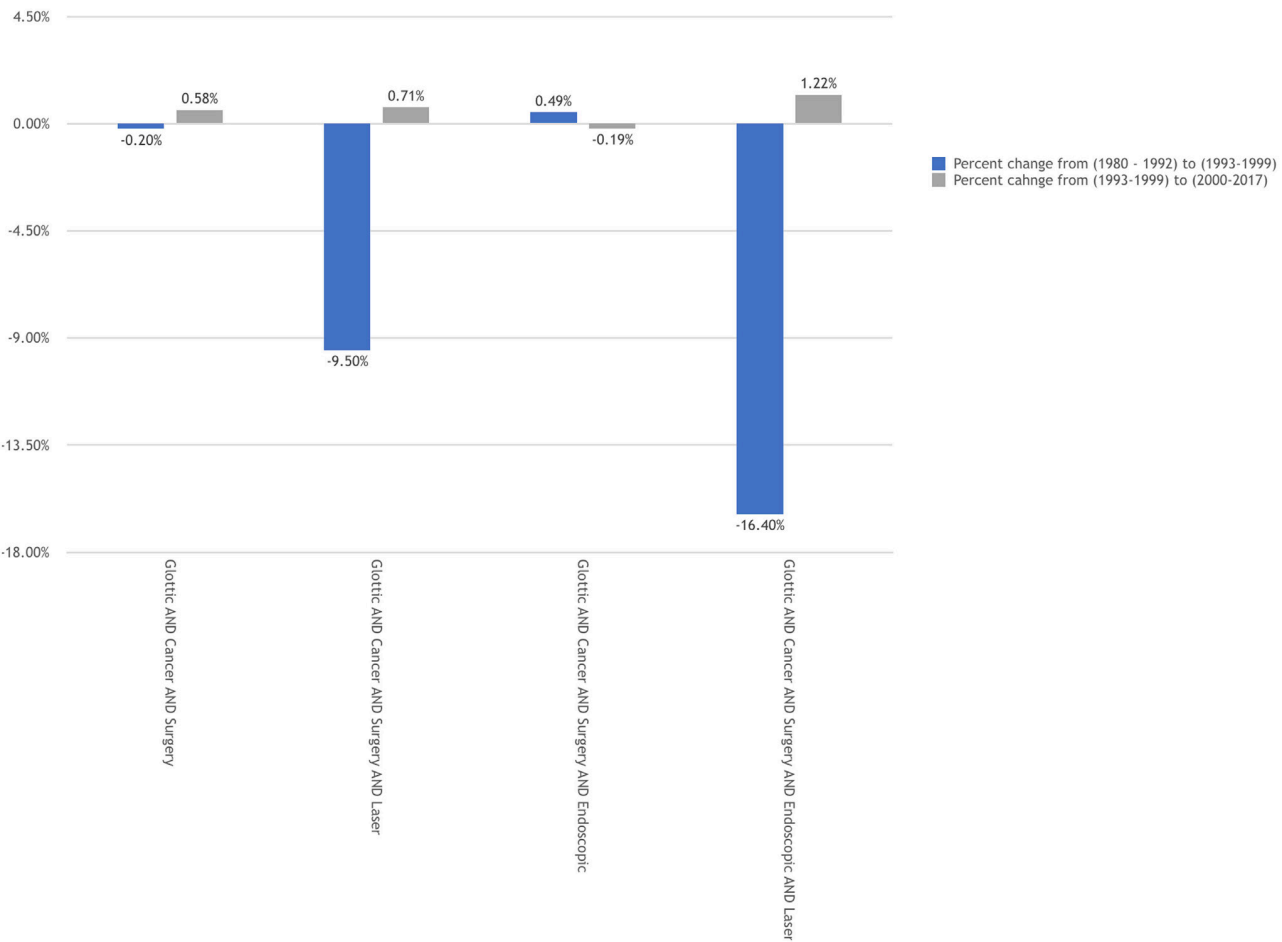
Corrected annual growth rates by time period for "GLOTTIC CANCER" publications.

**Figure 4:**
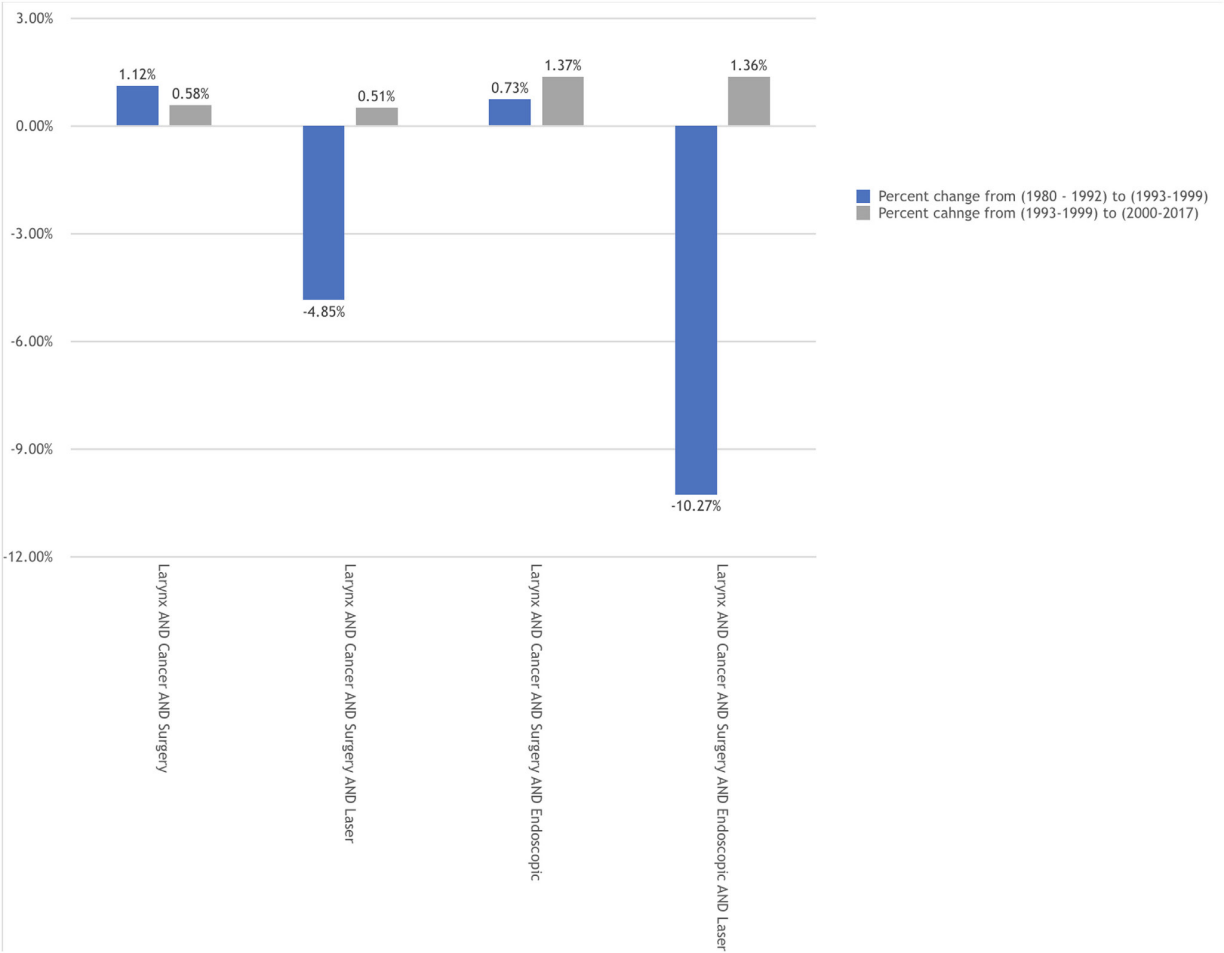
Corrected annual growth rates by time period for "LARYNX CANCER" publications.

The goal of the analysis was to evaluate the effects of the two landmark manuscripts on the publication rates. To assess this effect, the change (delta) in the corrected annual publication growth rates are displayed in Figures 3 and 4. When comparing the two time points (1993 versus 2000) we see that three of the four search groups for each search title maintained a larger annual growth rate in the period following the ELS classification scheme. Interestingly, several of the search groups demonstrated a negative change in the annual growth rate comparing prior to versus following 1993. This negative growth rate change was only seen in one search group and at a very slight negative rate (−0.19%) for that single group.

## Discussion

We sought to evaluate the effect of two landmark publications in the field of surgery for glottic cancer. Publication rates were chosen as the effect of interest in this study. However, a side-by-side comparison of publication rates between the time periods of interest results in skewed analysis in that publication rates for glottic cancer overall is not stable during these time periods. As overall medical journal publication rates can vary substantially, as well as publications covering the entire topic of throat cancer, the smaller subset of publications regarding the surgical care within our area of concern would be likewise affected. As such, the present analysis was adjusted to correct the overall change in publications over these time periods for the general area of interest, both glottic cancer as well as larynx cancer.

Utilizing a correct CAGR, we first calculated the growth rate of publications within the topics of glottic and larynx cancer covering endoscopic/laser/surgery prior to Professor Wolfgang Steiner’s landmark study describing his experience. We then calculated the corrected rates between Steiner’s work and publication of the ELS classification scheme of endoscopic laser cordectomy. Finally, we calculated the correct annual publication growth rate for the time period following the ELS paper. We demonstrated a substantial increase in the publications on endoscopic/laser/surgery as compared with the time periods prior.

An erroneous conclusion would be to create a direct comparison between the importance of these two landmark studies, somehow considering one manuscript superior than another. In point of fact, a comparison of importance would be a wasteful exercise without value. Instead, what this analysis set out to examine is the overall dispersion and propagation effects of the studies of interest. Stated in the form of a question, which work offered a wide acceptance rate of endoscopic laser surgery for glottic cancer? The value of such a question is to investigate the influencing factors of advancing surgical techniques to provide the guidance for the upcoming future surgical advances.

Steiner’s publication describing a technique so aggressively bucking the accepted oncologic dogma that its resulting aftershocks were felt for years following. Though initially met with heavy skepticism and outright rejection, Steiner’s undeniable success combined with his vast patient experience ultimately provided the foundation for the technique as it exists today. However, as demonstrated in the current analysis, glottic cancer patients outside of Göttingen were unlikely to reliably have this surgical option available to them. We may only theorize the reasons as why Steiner’s proven successful techniques were not being practiced and published with regularity. Some would suggest that their success rates were not readily replicated. Others would suggest that the anatomic and surgical complexity in which the piecemeal resections required left few surgeons with the confidence to promote the approach.

In fact, the variability of the transoral laser microsurgery techniques being described in the 1980s and 1990s was a major motivating factor for the ELS to formalize their recommendations. In quoting from their original manuscript, “We believe that non-standardized surgery, which requires years of training to understand its limits, offers little reproducibility to the majority of laryngologists.” Herein, we propose that the analysis gives credence to the ELS original goals of creating a technique and treatment option which offers wide application.

When reviewing the literature on the topic we have seen several authors to investigate the current practice trends for the treatment of glottic cancers utilizing one of several methods. First would be to query national patient databases by diagnosis (10, 11). However, while such large-scale databases offer substantial sample size analysis, the dependence on surgical coding leaves very much to be assumed regarding actual surgical techniques utilized. A second approach has been surgeon survey studies (12, 13). Even in the published studies which boast a high level of surgeon participation and response, these studies suffer from dependence on memory bias and assumption.

The present analysis here too relies on assumptions, mainly that all the publications included in the calculations are actually relevant to the topic instead of artifacts of search algorithms. This concern was deemed manageable in the authors’ opinion as the publication numbers, and by extension the publication growth rates, were compared from group to group and, therefore, any artifacts presents would be canceled out during the comparative calculations. The second assumption was that publication rates in and of themselves stand as a proxy for the adoption of endoscopic laser surgery for glottic cancer. Admittedly this consideration requires a jump when considering effect and causation; however, in the authors’ opinion this is a relatively small jump especially when considering the limitations of the alternative investigative strategies. There are several possible alternative explanations of the results of the data analysis including increases due to an undetermined long learning curve for the techniques with subsequent underreporting of results in the early years as well as increased attention for the technique during otolaryngology conferences.

## Conclusion

After comprehensive review regarding the endoscopic laser surgical treatment of glottic cancer, we have demonstrated that scientific publications increased in frequency following the European Laryngological Society (ELS) Cordectomy Classification proposal. This increase was beyond the changes seen in publication seen over a decade earlier following Professor Steiner’s landmark paper on the laser resection of glottic cancer. We conclude that the standardization of advanced surgical techniques as seen with the ELS classification scheme improves widespread adoption leading to increased patient access.

## Author Contributions

AM was involved in research planning, data acquisition, and manuscript organization. MR was involved in research planning, supervision, manuscript revision, and oversight.

## Conflict of Interest Statement

The authors declare that the research was conducted in the absence of any commercial or financial relationships that could be construed as a potential conflict of interest.
